# Feasibility of proton daily QA prototype for pencil beam scanning carbon ion beam therapy

**DOI:** 10.1002/acm2.70502

**Published:** 2026-02-12

**Authors:** Matthias Witt, Veronika Flatten, Andreas Schönfeld, Kilian‐Simon Baumann, Uli Weber, Klemens Zink

**Affiliations:** ^1^ Institute of Medical Physics and Radiation Protection, University of Applied Science Gießen Germany; ^2^ Department of Radiotherapy and Radiation Oncology Marburg University Hospital Marburg Germany; ^3^ Marburg Ion‐Beam Therapy Center (MIT) Marburg Germany; ^4^ SunNuclear, a Mirion Medical Company Melbourne Florida USA; ^5^ Biophysics Division GSI Helmholtzzentrum für Schwerionenforschung Darmstadt Germany

**Keywords:** carbon ion therapy, daily QA, PBS QA

## Abstract

**Purpose:**

The complexity of beam delivery and the limited availability of dedicated quality assurance (QA) devices present unique challenges for carbon ion beam therapy. Most existing systems are designed for single‐parameter verification and have been adapted from photon or proton workflows, resulting in time‐consuming QA procedures. This study aimed to evaluate the feasibility of using a proton QA prototype device (DQA‐P) in a pencil beam scanning (PBS) carbon ion facility, with the aim of enabling fast, efficient, multiparameter QA.

**Methods:**

The DQA‐P integrates 25 ionization chambers at three water‐equivalent depths, enabling the simultaneous measurement of multiple beam parameters, such as dose output, range, spot size, and spot position, in accordance with the recommendations of the AAPM TG‐224. Over 60 measurement sessions were performed, including 47 sessions involving intentional deviations in individual beam parameters, in order to assess sensitivity. The measurements were analyzed retrospectively using custom MATLAB scripts to evaluate accuracy, reproducibility, and the impact of setup uncertainties.

**Results:**

The DQA‐P prototype successfully measured all QA parameters within clinically acceptable tolerances. Dose output measurements showed a standard deviation of 0.5% around the mean, with minimal systematic deviation. Spot position measurements exhibited a mean deviation of 0.16 mm, with a standard deviation of 0.32 mm. The device's sensitivity was demonstrated by its reliable detection of intentional positional offsets of ±3 mm and spot size changes of ±25%. Range validation using the proximal rise of the Bragg peak produced consistent results, albeit with lower accuracy than that achieved with standard multilayer ionization chambers. However, limitations were identified in spot size estimation due to chamber segmentation, and in range verification, as the device only captures three fixed depths.

**Conclusions:**

The DQA‐P prototype demonstrated feasibility for daily QA in PBS carbon ion beam therapy, offering a short overall measurement time of approximately 5 min, efficient data acquisition, and simultaneous multiparameter verification within a single setup. This is a significant improvement on the conventional daily QA procedure, which uses two different detector types for independent dose and spot position measurements. In the standard workflow, both detectors must be positioned individually using the in‐room laser system, connected to the readout electronics and irradiated using the daily beam plan. Altogether, this procedure takes around 15–20 min. Although certain limitations remain—particularly in spot size accuracy and range determination—the device showed sufficient sensitivity and stability for daily QA purposes. Its use has the potential to streamline QA procedures, reduce beam time, and enhance workflow efficiency in carbon ion therapy.

## 1 INTRODUCTION

Quality assurance (QA) is essential in all forms of radiotherapy to ensure that the correct dose is delivered safely and accurately to the patient. However, the complexity and number of adjustable parameters are significantly higher in particle beam therapy, especially in carbon ion therapy, compared to photon therapy. The complex physical and biological properties of carbon ions, such as their sharp Bragg peak and high linear energy transfer (LET), mean that QA requirements are more demanding than for conventional photon or even proton therapy. Despite these challenges, and given that only a few centers offer carbon ion therapy,^1^ there are only a limited number of QA devices specifically designed for carbon ion therapy. Most available systems have been adapted from proton or even photon QA workflows and are usually optimized to assess only one parameter, such as range, dose output, or spot position.^2^, ^3^, ^4^

This fragmented approach results in longer QA times and an increased workload, as multiple setups and devices are required to verify the full set of beam parameters recommended by international guidelines, such as the AAPM TG‐224 report.^5^ In high‐throughput clinical settings, QA time is a critical resource. Reducing this time while maintaining or improving QA sensitivity is therefore of great interest.

One promising strategy is to develop and implement multifunctional QA devices that can measure several beam parameters, such as spot size, position, range, and dose output, in a single irradiation and setup. Such integrated systems can streamline the QA process, improve workflow efficiency, and enable comprehensive monitoring of beam quality. While these devices are commonly available in photon therapy to enable a fast, secure, and streamlined daily QA routine, they are lacking in carbon ion therapy.

The Daily QA Proton array (DQA‐P, SunNuclear, Florida, USA) is a prototype device that was originally developed for daily QA tasks in proton beam therapy. An extensive study has already been performed to assess its feasibility in proton beam therapy QA.^6^ This study evaluates the feasibility of using the DQA‐P in a pencil beam scanning (PBS) carbon ion therapy facility. The device's distinctive design features multiple ionization chambers arranged at various water‐equivalent depths, enabling the simultaneous assessment of multiple beam parameters. Combined with the inherent robustness and stability of ionization chamber technology, the DQA‐P shows promise as an efficient and reliable tool for daily QA in carbon ion therapy. Its ability to integrate multiple measurements into a single setup has the potential to streamline QA procedures and reduce QA time and uncertainties in setup, making the DQA‐P a strong candidate for routine clinical use in complex particle therapy environments.^6^

A key focus of the study was to determine whether the distinct features of carbon ions could be accurately measured using the DQA‐P. Specifically, the following questions were addressed: Can the smaller spot sizes of carbon ions be reliably measured using segmented ionization chambers? Does the higher LET of carbon ions cause quenching effects? Can the sharper Bragg peak of 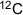 be accurately assessed? Does the fragmentation tail interfere with measurements? Each of these issues was investigated, and coping strategies were presented where limitations were identified.

## 2 METHODS

### 2.1 Proton daily QA prototype

The prototype device used in the study was provided by Sun Nuclear (Sun Nuclear, A Mirion Medical Company, Florida, USA) and is designed for fast measurement of daily QA procedures on proton machines, hence its name DQA‐P. A detailed description of the array can be found in Flatten et al.^6^

**FIGURE 1:**
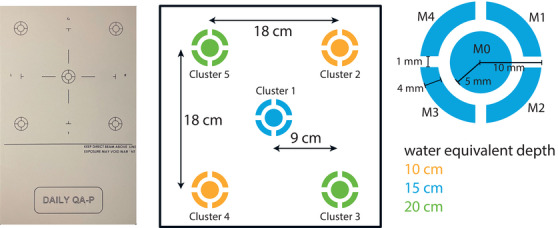
Image and schematic details of the DQA‐P array prototype. On the left is a frontal view. The position of the ionization chambers arranged in clusters is visualized in the center. The colors correspond to the water equivalent depth of the chambers (shown at the bottom right). A detailed view of each ionization chamber cluster is shown on the right. The total size of the array is 432 mm × 260 mm × 36 mm.

The array is shown in Figure 1, providing a schematic of the chamber positions and arrangement. It consists of ionization chambers organized into clusters, with each cluster positioned at a specific water equivalent depth. The water equivalent depths of 10, 15, and 20 cm are achieved using tungsten as the range‐degrading material. This unique design enables the measurement of various beam characteristics in alignment with international protocols, such as the AAPM TG‐224 Report.^5^ Table 1 lists the relevant beam dosimetry parameters along with their recommended tolerance levels.

**TABLE 1:** Dosimetric tolerances for daily QA procedures according to AAPM TG‐224 Report.^5^

Procedure		Tolerance
Output constancy		± 3%
Range verification	Distal	± 1 mm
	Proximal	± 2 mm ^a^
Spot position	Absolute	± 2 mm
	Relative	± 1 mm
Spot size		± 10% ^b^

^a^ Tolerance for uniform scanning instead of PBS.

^b^ Recommended monthly or annual QA procedure.

The output constancy can be assessed directly as the array consist of ionization chambers. With built‐in sensors, an automatic temperature and pressure correction $k_{\mathrm{TP}}$ is applied, therefore the reading of the ICs directly provides the dose output. Measurement of all parameters other than the dose output requires a dedicated calibration procedure as described in Section 2.3. A list of all measurement parameters tested in this study can be found in Table 1.

### 2.2 Marburg Ion Beam Therapy Center

All measurements were performed at the Marburg Ion Beam Therapy Center (MIT, Marburg, Germany).^7^ At MIT Marburg, carbon ions can be accelerated from 86 MeV/u up to 430 MeV/u, corresponding to ranges between 2 and 32 cm in water, respectively. The spot size at the isocenter, located 1.4 m downstream of the beam exit window, ranges from 13.5 to 3.4 mm full‐width at half maximum (FWHM). In comparison, the spot size at isocenter for typical proton machines (e.g., the IBA Proteus ONE) ranges from 18.8 to 7 mm for energies between 70 and 226 MeV.^8^ At MIT Marburg, the proton spot sizes range from 32.5 to 9.8 mm for energies ranging from 48 to 220 MeV.

### 2.3 Calibration procedure

**FIGURE 2:**
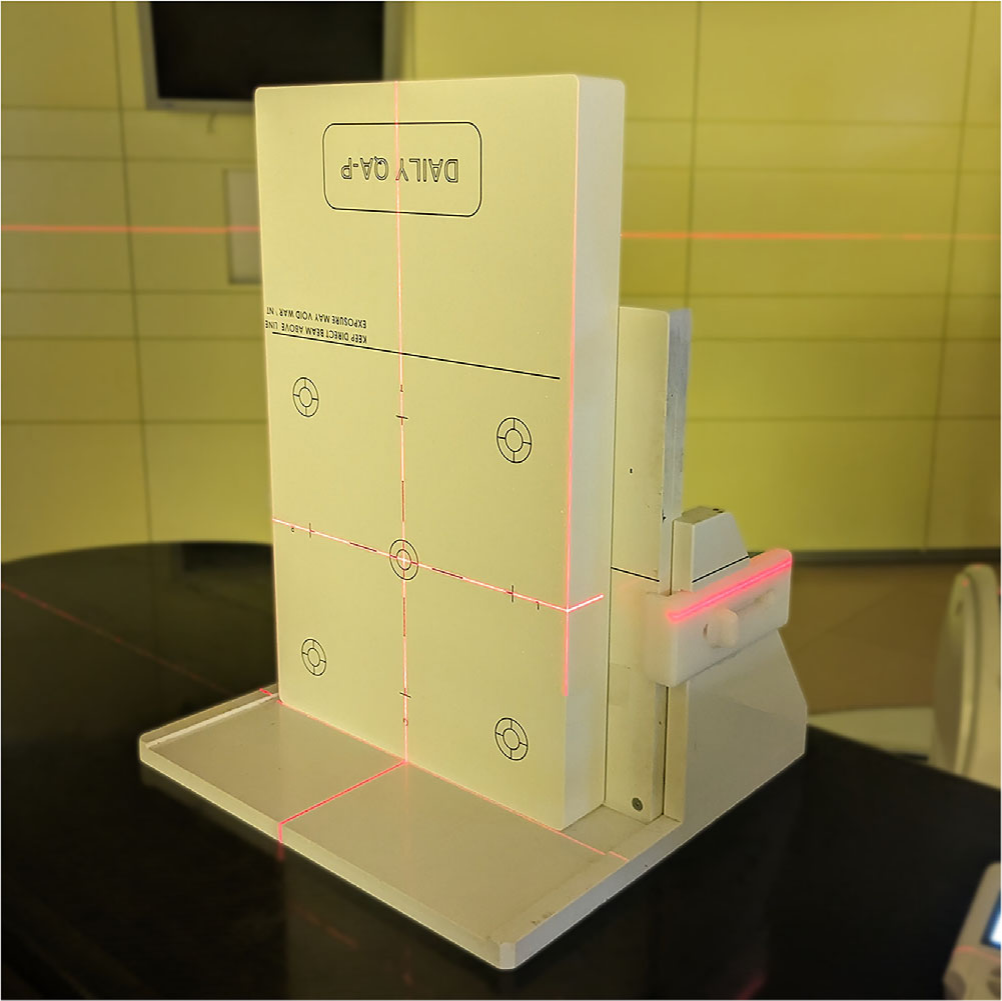
DQA‐P setup of for an irradiation in a room with a fixed horizontal beam line. The connection sockets are located at the bottom of the array, therefore the array is upright and upside down. The device is attached to a support to ensure that it is positioned safely.

The calibration procedure is performed using the same setup as for the daily measurements. The central chamber cluster (Cluster 1) of the DQA‐P is positioned with its surface point at the isocenter with the help of the in‐room laser system. At MIT Marburg, where only fixed beamlines are available, the array is placed upright and upside down (see Figure 2) due to the location of the connection plugs at the bottom of the array. Before calibration and daily measurements, the dose linearity of the ionization chambers was verified.^6^ For consistency, the term “range verification” is used throughout this work, where the terms “beam energy” and “range” are used interchangeably.

**Spot position calibration**: The spot position can be assessed for each of the five clusters. The calibration procedure is performed by delivering a specially designed calibration beam plan. A scan pattern with positional offsets ranging from 0 to ±4 mm was applied and measured. For each positional offset, the signal change of two opposing chambers (see Equations 1 and 2) is calculated for each direction independently (horizontal (*x*) and vertical (*y*) in beam's eye view).

(1) $$A_{x}=\frac{2(M1+M2)}{\sum_{i=1}^{4}M_{i}}$$

(2) $$A_{y}=\frac{2(M1+M4)}{\sum_{i=1}^{4}M_{i}}$$

**FIGURE 3:**
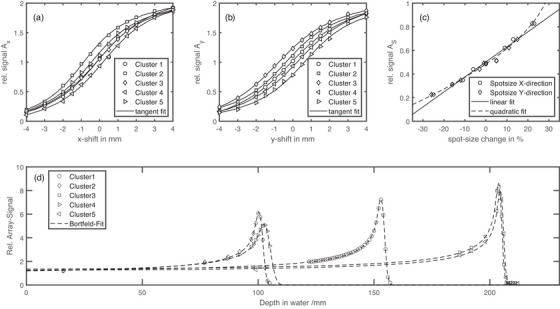
Result of the calibration procedure. The spot position in horizontal (a) and vertical direction (b) and the spot size (c) are shown. A predefined beam pattern was irradiated with positional shifts of ±4 mm. The array signals $A_{x}$ and $A_{y}$ are computed according to Equations (1) and (2). For the spot‐size, five beam spots of the same energy (430 MeV/U) and different spot‐size of up to ±25% were measured and the array signal $A_{s}$ is computed by Equation (3). For the spot position, a tangent fit, and for the spot‐size, a linear fit and a quadratic fit is used to estimate the differences in the daily beam plan irradiation. The range‐calibration measurements for all five clusters are shown in (d). By increasing the energy of the accelerator in range steps, corresponding to 1‐mm depth in water, the Bragg peak can be measured. The dashed line displays the result of the Bortfeld‐fit.

With $M_{i}$ being the chamber number according to Figure 1. Figures 3a and 3b show the measurement results for the spot size calibration. The signal change depending on the positional offset for each cluster can be described by a tangent fit. This tangent fit is stored in a baseline to compute the positional offsets for daily beam plan irradiation.

**Spot size calibration**: The spot size can also be assessed for each of the five clusters. Five spots with the same primary beam energy and dose but varying spot sizes (±25%) are applied and measured. The change in signal of the sum of the outer chambers (M1–M4) relative to the central chamber (M0) is calculated (see Equation 3).

(3) $$A_{s}=\frac{\sum_{i=1}^{4}M_{i}}{M0}$$

With $M_{i}$ being the chamber number according to Figure 1. Figure 3c shows the signal change as a function of the applied spot size for the central cluster (C1). Both a linear and a quadratic fit were used to describe the signal change. These fits are stored in a baseline to calculate the difference in spot size in the daily beam plans.

**Dose output**: The central chamber (M0) of each cluster was used to assess the dose output. Small, mono‐energetic fields (20×20 ${\mathrm{mm}}^{2}$) were applied to minimize sensitivity to positional or setup errors. The dose output was determined from the relative signal change of chamber M0. Flatten et al. demonstrated that the change in the array signal is directly proportional to the delivered dose.^6^ The reference signal for each chamber M0 in Clusters C1–C5 was stored in the baseline and the relative difference was calculated to estimate dose variation during daily beam plan irradiation.

**Range verification**: To verify the energy or range, small mono‐energetic fields (20×20 ${\mathrm{mm}}^{2}$) were also irradiated and measured at the central chamber M0. Subsequently, multiple fields with varying energies, ranging from the maximum energy of 430 MeV/u down to the energy at which the beam stops just before the chamber, were irradiated. The accelerator energy was varied incrementally, corresponding to discrete 1‐mm water‐equivalent depth shifts. At each energy, the signal from the central chamber (*M*0) was measured, and the relative depth–dose curve was reconstructed across the fixed water‐equivalent depths of the DQA‐P array. This approach effectively samples the Bragg peak without mechanical depth scanning. Depending on the amount of degrading material in front of the chamber cluster, the energies are 281 MeV/u for C3 and C5, 220 MeV/u for C1, and 146 MeV/u for C2 and C4. During the calibration process, the relative signal is calculated as the quotient of the measured signal of Chamber *M*0 for each energy, divided by the signal of Chamber *M*0 for the highest energy. This establishes a relative depth dose curve. Then, a Bortfeld‐fit without fragmentation tail (see discussion section 4) is performed on the measured Bragg curve.^9^ The obtained Bortfeld‐fit is stored in the baseline and used to calculate range differences based on the signal changes.

The entire calibration process for a synchrotron‐based machine takes around 10 min of beam time. The most time‐consuming part is the range mapping for each cluster, given that the acceleration cycles at the MIT Marburg facility take around 3 s per energy change. Therefore, depending on the number of energies irradiated, the total irradiation time can vary. For the central cluster, for which detailed measurements of both the proximal and distal regions were performed, the required beam time was approximately 5 min. Once calibration has been completed, it is not necessary to recalibrate for routine daily QA measurements over extended periods.

### 2.4 Daily beam plan irradiation

After the calibration process is performed, a different beam plan is irradiated and evaluated for each beam parameter. The daily beam plan consists of three parts. First, the range is tested by irradiating a small mono‐energetic field (20 × 20 ${\mathrm{mm}}^{2}$) at each cluster position. This energy is specific to each cluster, depending on the amount of degrading material in front of it. The energy is chosen so that the measurement depth of the chamber is at the proximal rising edge of the Bragg peak. The following energies were used for Clusters 1–5: C1: 235 MeV/u; C2 and C4: 151 MeV/u; C3 and C5: 288 MeV/u. In the second part, the spot position and size are tested by applying a single spot of the highest energy (430 MeV/u) to the center of each cluster. In the final part, the dose output was measured by irradiating a small, mono‐energetic field (20 × 20 ${\mathrm{mm}}^{2}$) with the highest energy to the center of Cluster 1. This energy was chosen to be robust against differences in energy as the measurement depth is in the plateau region of the Bragg peak. A total of 60 measurement sessions were conducted, including 13 without intentional errors. To assess the device's sensitivity in detecting errors, 47 measurements were carried out with intentional errors. For these measurements, at least one beam quality parameter (spot size, spot position, beam range, or delivered dose) was deliberately altered. The measurements were performed over a period of 4 months; during this time, the calibration measurement remained valid and a recalibration was unnecessary. The total time required for a complete daily QA measurement, including positioning using the in‐room laser system, connection of the device, and execution of the daily beam plan, was approximately 3–5 min. No additional phantom exchange or detector repositioning was necessary, as all beam parameters were evaluated during a single setup and irradiation. This substantially reduces QA time compared to conventional daily QA workflows, which typically require multiple devices and sequential measurements to dose and spot position.

## 3 RESULTS

With the established calibration, the daily beam plans can be irradiated and assessed for the daily QA parameters spot position, spot size, and dose output.

**FIGURE 4:**
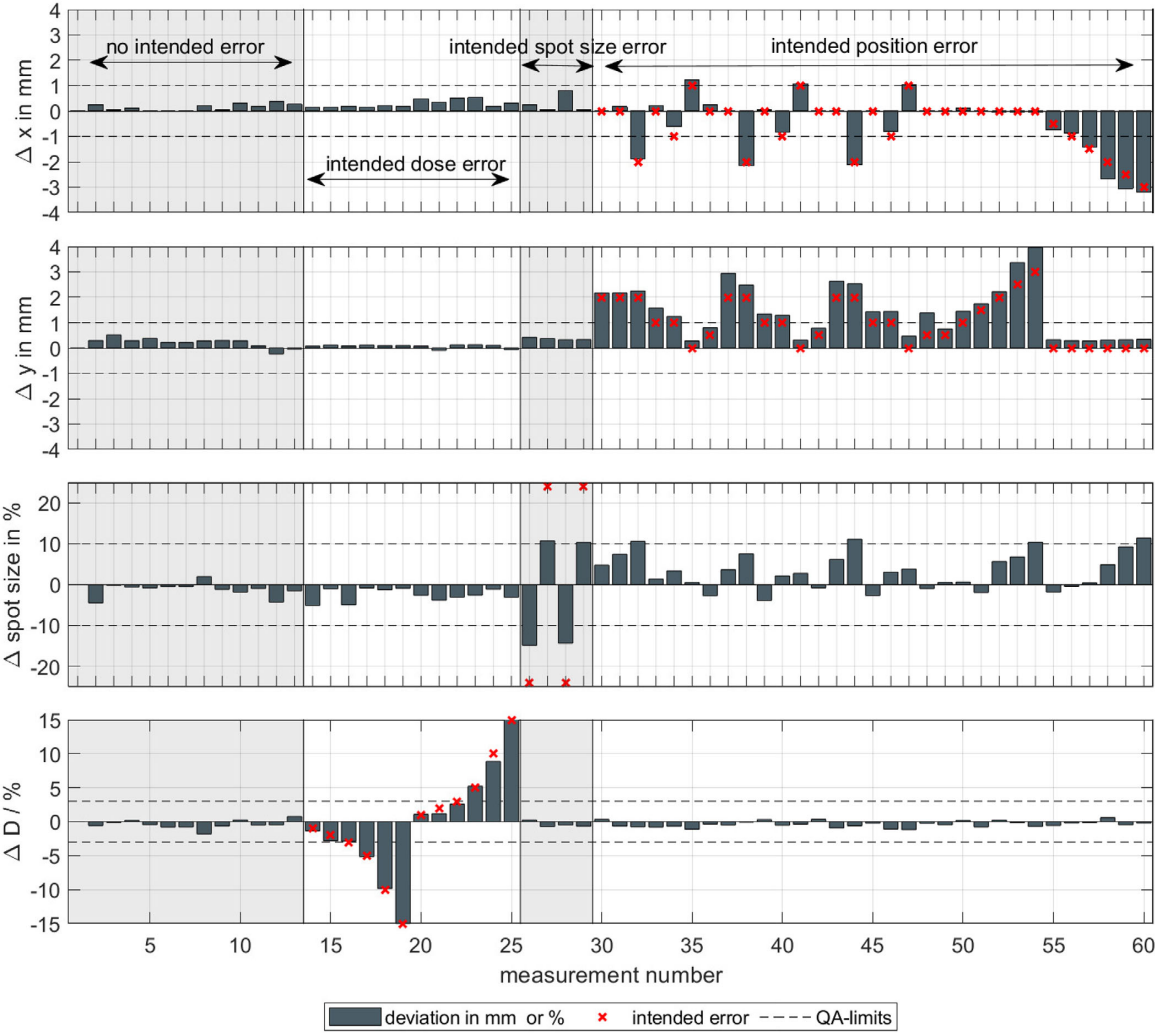
A total of 60 daily measurements taken over the period of several months are displayed, limited to the central cluster (C1) for visual clarity. The top two rows show the measured spot position deviation in millimeter in the horizontal ('x') and the vertical ('y') directions. The third row shows the measured spot size difference in percent. The last row shows the measured output difference in percent. The differently colored regions visualize the areas where the beam parameters were intentionally altered to test the sensitivity of the DQA‐P prototype. The first 13 measurements have no alterations, followed by 12 measurements with intended dose output errors and then four measurements with the spot size alterations. The final 31 measurements show deviations in the x‐ and y‐directions, or a combination of both.

**FIGURE 5:**
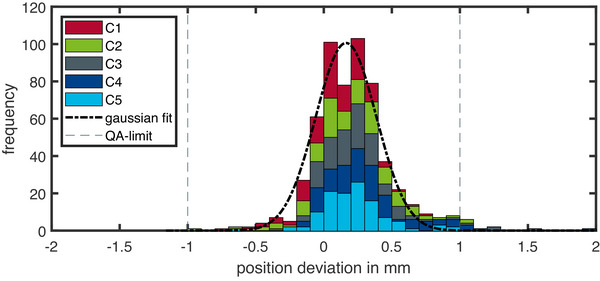
Results of the measured spot position deviation (in the *x*‐ and *y*‐directions) for all clusters (C1–C5) and all 60 measurements. The different bar colors correspond to the different clusters. The mean deviation for all clusters was measured at 0.16 mm, with a standard deviation of 0.31 mm. Of the 300 measurements, only 13 showed a deviation above the QA limit.

In Figure 4, the results for 60 QA measurement sessions are shown. To check the sensitivity of the DQA‐P device on alterations, in addition to 13 measurements without errors, 47 measurements were performed with deliberately altered beam parameters. For the 13 measurements with no alterations in the beam plan, all measured parameters are well within the daily QA limits. The maximum observed changes were 0.8 mm for spot‐position, approximately 2.5% for spot‐size and approximately 1.6% for the output. For deliberately altered spot‐size of ±25%, an underestimation of the measured spot size was found if a linear fit is used. These large variations were introduced intentionally to evaluate the device's sensitivity and robustness, and do not represent clinically acceptable conditions. In clinical operation, such deviations would be detected and corrected well before reaching this magnitude through routine machine QA and beam tuning procedures. For smaller spot sizes, an underestimation of approximately 10% was found, and for larger spot sizes, approximately 15% was found. Using a quadratic fit to calculate the spot size (see Figure 3) reduces the calculated deviations to 7% and 6% for larger and smaller spot sizes, respectively. If the spot position is changed by up to ±3 mm, the DQA‐P reproduces the positional changes within 0.6 mm. However, positional errors were found to influence the spot size, with offsets exceeding 3‐mm result in spot size errors above the proposed QA limit. The design of the daily beam plan allows to check the spot position for each of the five clusters. The results of all spot position measurements for every cluster are shown in Figure 5. When all clusters are evaluated for spot position, a mean deviation of 0.16 mm with a standard deviation of 0.31 mm is found. It is important to note that these values represent the system's ability to reproduce the spot position relative to the baseline calibration. While the device's absolute positioning accuracy is typically limited to ±0.5 mm by the in‐room laser system, the low standard deviation demonstrates the DQA‐P array's intrinsic precision and stability. For daily QA purposes, the primary goal is to detect deviations from the baseline rather than absolute dosimetry, so this reproducibility is the critical metric. While laser alignment uncertainty is statistically distributed, the system consistently detects spot positions with sub‐millimeter precision. A total of 300 measurements can be evaluated, 95% of which are found to be within the QA limit.

**FIGURE 6:**
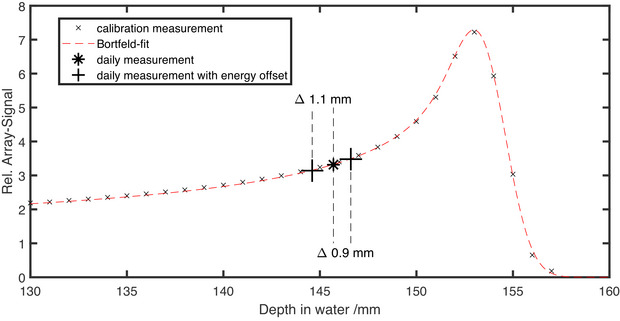
Result of the range check in the proximal part of the Bragg peak for the central cluster (C1). The crosses represent the calibration measurement with the Bortfeld‐fit shown as dashed line. Daily measurements were performed at the marked range (asterisk), and additional measurements with an intended range shift of ±1 mm are shown as pluses. The range shifts measured and computed with the Bortfeld‐fit are 1.1 and 0.9 mm, respectively.

The final parameter investigated was the beam range of the accelerator. For this purpose, the energy of the accelerator was intentionally altered for the central cluster (C1). The minimal energy change corresponds to a range change of 1 mm in water. The results of the measurements are shown in Figure 6. The difference calculated using the established Bortfeld‐fit between the intended and the measured range shift was found to be 1.1 and 0.9 mm, respectively.

## 4 DISCUSSION

In summary, the DQA‐P prototype could measure all the quantities specified in AAPM TG‐224 (see Table 1). However, limitations were observed when using the device for carbon ion measurements. Dose measurements with the DQA‐P exhibited a standard deviation of 0.5%, which is comparable to that of the standard dose measurement method at MIT Marburg. The minimum and maximum deviations measured were +1.6% and −1.05%, respectively, which are slightly higher than expected. For measurements involving intended output errors, introduced by altering the number of ions in the beam plan, the observed deviations were below −1.1%, with a mean deviation of −0.2%. No correlation was observed between larger intended errors and increased measured deviations. For comparison, the standard dose measurement method at MIT Marburg employs an ionization chamber (PTW, Freiburg, Farmer chamber TM30013). Here, the dose is measured at the center of a 60 × 60 × 30 ${\mathrm{mm}}^{3}$ dose cube at a water depth of 6 cm. The standard deviation for this method is also approximately 0.5%, which is consistent with the DQA‐P measurements. However, the minimum and maximum observed deviations are smaller. Unlike the standard method, DQA‐P enables five dose measurements to be acquired at three different depths (C1–C5) without changing the setup.

On average, the measured spot position across all clusters and measurements was 0.16 mm, with a standard deviation of 0.32 mm. The maximum deviations ranged from –1.3 to 2.1 mm. A slight trend was observed, indicating that larger intended positional offsets tended to result in greater deviations. Of the 300 measurements, only 13 exceeded the QA tolerance, all of which were located in the outer clusters (C2–C5). In these cases, the beam is deflected, and small setup errors—particularly rotational misalignments of the DQA‐P along the beam axis—can amplify deviations. Assuming a maximum rotational misalignment of 0.5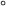, we conservatively estimate a positioning uncertainty of 0.8 mm for the clusters C2–C5. For the central cluster (C1), we conservatively assume a maximum positioning uncertainty of 0.5 mm, which is primarily attributed to the limited setup accuracy achievable using in‐room laser alignment. Consequently, measured positional offsets below this threshold cannot be unambiguously attributed to either intrinsic beam offsets or true positional deviations. This limitation is inherent to any laser‐based daily QA setup and is not specific to the DQA‐P system. This additional uncertainty is taken into account in the evaluation of the measured spot positions. In this study, alignment was performed using device markings and the in‐room laser system, without making use of the built‐in fiducial markers. Employing in‐room imaging for more accurate positioning could likely reduce these deviations. The standard procedure for measuring spot position and spot size at MIT Marburg employs a multiwire chamber (Siemens Healthineers, Erlangen).^10^ With this method, positional deviations are typically below 0.5 mm, which is in good agreement with the measurements obtained using the central cluster C1 of the DQA‐P. For deflected beam spots, similar deviations are observed, but an increased QA tolerance is applied. An additional difference between the two methods is the accessible measurement depth. With the multiwire chamber, spot size can be assessed at very shallow water‐equivalent depths, whereas in the DQA‐P, the minimum measurement depth corresponds to 10‐cm water. As a result, the measured spot size in the DQA‐P is partially influenced by scattering effects in the degrading material, and the contribution from the optical magnetic focusing of the beam line is correspondingly superimposed. For the spot size, an underestimation of approximately 10% for larger spot sizes and 15% for smaller spot sizes was observed. Although spot size is not explicitly recommended for daily QA in AAPM TG‐224, it was included in this study as a consistency check to monitor beam focusing stability in carbon ion therapy. The intention is to detect trends rather than determine the absolute spot size. Additionally, greater deviations were observed in the evaluation method if the spot position differed from the origin. For positional offsets greater than ±3 mm, the estimated spot size exceeded the daily QA tolerance. Spot size determination was based on a linear fit of the array signal, in accordance with the method outlined by Flatten et al.^6^ In the context of daily clinical QA, the purpose of spot size evaluation is to detect deviations from baseline behavior, rather than quantifying large absolute changes, which would be identified well before they reach a level of clinical relevance. While this approach was effective for protons, a quadratic fit better represented the carbon ion data (see Figure 3). This reduced deviations to within 7%, which is in line with the daily QA limits. The standard measurement procedure for spot size also utilizes the multiwire chamber.

For the range verification, Flatten et al.^6^ used the distal fall‐off of the Bragg peak for protons. However, carbon ions have a much steeper distal fall‐off, in comparison, the distal 80%–20% fall‐off for the highest carbon energy is 1.8 mm, whereas for the highest proton energy, it is 4.4 mm. If the distal 50% fall‐off is used as reference point, a range shift of 1 mm will result in measurements being taken in the fragment tail or ambiguous results will be obtained from the rising or falling edge of the Bragg maximum. Therefore, the measurement principle in combination with the steep distal fall‐off proved too unstable for use. Instead, the proximal part of the Bragg peak was used to validate range. A Bortfeld‐fit was used to calculate the range difference based on the signal change as described in Section 2.3. Although the Bortfeld‐fit does not include the fragment tail of heavier ions, the other parts of the curve are well described and have been proven to provide reliable results for tested range changes in the proximal part of the Bragg peak (see Figure 6). Additionally, since we use the proximal part of the Bragg peak for range analysis, the fragment tail does not interfere with the measurement data. In contrast, if the distal part is used, a range shift measured in the fragment tail region of the Bragg peak cannot be adequately analyzed, as the signal change is very small. Standard methods for verifying the range in ion beam therapy include multilayer ionization chambers (MLICs) and 1‐D water columns,^3^, ^11^, ^12^, ^13^ both of which capture the full Bragg peak. While 1‐D water column measurements can take several minutes depending on resolution and accelerator type, MLICs require only a single irradiation. In contrast, the presented method measures dose at three discrete depths, rather than capturing the full depth‐dose curve. Despite this limitation, it offers a quick and practical additional check of beam range at no extra cost, making it a suitable option for daily QA. The range shift of the Bragg peaks visible in Figure 3d for Clusters C2 and C4 was reproducible. The observed shift of approximately 3 mm corresponds to a difference in solid tungsten material of roughly 150 μm, which is consistent with the specified manufacturing tolerances.

All data in this study were analyzed retrospectively, as the current prototype of the DQA‐P did not include software for immediate analysis or visualization. Measurement results were processed using a custom MATLAB script (MathWorks Inc., Massachusetts). As a result, observed deviations could not be verified or corrected in real time, and potential setup errors or misalignments could only be identified after the measurement session. The DQA‐P prototype offers significant potential benefits for daily QA in particle beam therapy. Its unique design enables the simultaneous measurement of all the key beam parameters recommended in the AAPM TG‐224 report, such as dose output, range, spot size, and spot position. This comprehensive capability enables efficient, streamlined QA processes. The array's compact design and intuitive handling allow for quick and reliable positioning at the isocenter. Setup can be facilitated using in‐room laser systems and markings or positioning devices with built‐in fiducial markers, which improve alignment accuracy and reproducibility. From a clinical workflow perspective, the DQA‐P makes the daily QA procedure for PBS carbon ion beam therapy substantially more efficient. By combining all the necessary measurements in a single setup, the total measurement time is reduced to around 5 min. In contrast, the conventional daily QA workflow relies on separate detector systems for dose and spot position verification, each requiring individual positioning with the in‐room laser system, connection to the readout electronics, and irradiation using the daily beam plan, resulting in a total measurement time of approximately 15–20 min. While certain limitations were identified—most notably in absolute spot size accuracy and range determination—the observed sensitivity and stability of the measured parameters are sufficient for daily QA applications. In this context, the DQA‐P should be regarded as a tool for consistency monitoring and early detection of systematic deviations rather than absolute beam characterization. Its implementation therefore has the potential to streamline daily QA procedures, reduce required beam time, and improve overall workflow efficiency in clinical carbon ion therapy.

The smaller spot sizes of carbon ions compared to protons required an adapted evaluation routine. Specifically, the determination of the spot size was modified to use a quadratic fit instead of the linear fit previously described by Flatten et al.^6^ Within the investigated clinical parameter range, no LET‐related effects on the measurement principle were observed. No LET‐ or dose‐rate‐dependent effects were observed under the specific measurement conditions applied in this study. However, a systematic investigation of dose‐rate dependence was beyond the scope of this study and was formerly performed by Flatten et al.^6^ Additionally, the sharper distal fall‐off and the presence of a fragment tail in the carbon ion Bragg peak did influence both the measurement setup and the evaluation routine. Consequently, range verification was performed in the proximal part of the Bragg peak instead of using the distal 50% fall‐off.

## AUTHOR CONTRIBUTIONS

Matthias Witt conceived the study, designed the comparison, collected and analyzed the data, and drafted the manuscript. Veronika Flatten contributed to the design of the analysis and substantially revised the manuscript. Andreas Schönfeld developed the analysis code and contributed to the study design. Kilian‐Simon Baumann contributed to the design of the analysis and substantially revised the manuscript. Uli Weber contributed to the design of the analysis and substantially revised the manuscript. Klemens Zink supervised the study, managed beam time access, and substantially revised the manuscript. All authors approved the final version of the manuscript and agree to be accountable for all aspects of the work.

## CONFLICT OF INTEREST STATEMENT

V. Flatten and A. Schönfeld are employed by SunNuclear.

## Data Availability

The data that support the findings of this study are available from the corresponding author upon reasonable request.
